# Immunomodulatory role of azithromycin: Potential applications to radiation-induced lung injury

**DOI:** 10.3389/fonc.2023.966060

**Published:** 2023-03-08

**Authors:** Yujie Yan, Leilei Wu, Xuefei Li, Lan Zhao, Yaping Xu

**Affiliations:** ^1^ Department of Radiation Oncology, Shanghai Pulmonary Hospital, Tongji University School of Medicine, Shanghai, China; ^2^ Department of Lung Cancer and Immunology, Shanghai Pulmonary Hospital, Tongji University School of Medicine, Shanghai, China; ^3^ Department of Respiratory and Critical Care Medicine, Shanghai Pulmonary Hospital, Tongji University School of Medicine, Shanghai, China

**Keywords:** radiation-induced lung injury, azithromycin, immunomodulation, inflammatory responses, mechanisms

## Abstract

Radiation-induced lung injury (RILI) including radiation-induced pneumonitis and radiation-induced pulmonary fibrosis is a side effect of radiotherapy for thoracic tumors. Azithromycin is a macrolide with immunomodulatory properties and anti-inflammatory effects. The immunopathology of RILI that results from irradiation is robust pro-inflammatory responses with high levels of chemokine and cytokine expression. In some patients, pulmonary interstitial fibrosis results usually due to an overactive immune response. Growing clinical studies recently proposed that the anti-inflammatory and immunomodulatory effects of azithromycin may benefit patients with acute lung injury. It has been shown potential benefits for patients with RILI in preclinical studies. Azithromycin has a variety of immunomodulatory effect to improve the process of disease, including inhibition of pro-inflammatory cytokines production participating in the regulatory function of macrophages, changes in autophagy, and inhibition of neutrophil influx. We review the published evidence of mechanisms of azithromycin, and focus on the potential effect of azithromycin on the immune response to RILI.

## 1 Introduction

Radiotherapy is an important treatment strategy for thoracic malignant tumors. However, as a radiation sensitive organ, the irradiation lung is prone to radiation-induced lung injury (RILI), including radiation-induced pneumonitis (RIP) and radiation-induced pulmonary fibrosis (RIPF), which severely reduces the efficacy of radiotherapy and affects the quality lives of patients (1). Severe RILI induced by irradiation showed a strong pro-inflammatory response in immunopathology, with various pro-inflammatory and pro-fibrotic cytokines from damaged and activated cells. In some patients, pulmonary interstitial fibrosis is usually caused by an overactive immune response. In addition, severe cases of acute RIP are characterized by cytokine storms and acute respiratory distress syndrome (ARDS) requiring immunosuppressive therapy. Cytokines are considered as important molecular factors involved in the signaling network in pathological processes. The clinical evidence and immunopathology of RILI show that radiation leads to changes in immune function in some individuals, resulting in hyperactive pro-inflammatory response. Some serious cases need to be treated with immunosuppressive therapies that can rebalance the immune system.

More and more evidences support macrolides like azithromycin (AZM) have anti-inflammatory and immunomodulatory properties. Mechanistic studies demonstrate immunomodulatory activity of AZM through the regulation of cellular processes involved in inflammation response *via* NF-κB signaling pathway (2, 3), inhibition of neutrophil influx, alteration of macrophage polarization (3), and autophagy flux (4, 5). Although azithromycin inhibits a variety of pro-inflammatory pathways, it will not lead to complete immunosuppression like glucocorticoids and other immunosuppressive therapies. In contrast, azithromycin shows immunomodulatory properties by transforming the inflammatory response dominated by macrophages into an inflammatory response characterized by the functional aspects of regulation and repair (6). These effects recall a profound effect for azithromycin on inflammatory conditions in which the immunomodulatory characteristics of macrolide antibiotics expand their therapeutic indications (7). Increasing data support the immunomodulatory effects of azithromycin on early inflammation, including inhibition of pro-inflammatory cytokine production, inhibition of neutrophil influx, induction of regulatory functions of macrophages, and alterations in autophagy (8).Tang et al. reported a study in which mice received irradiation followed by azithromycin. They concluded that azithromycin could ameliorate RILI through modulating the inflammation and fibrosis, especially in the high-dose group (9). The role of azithromycin in the treatment of RILI has also been a paramount concern for radiation oncologists. Here we review the published evidence of these mechanisms, and focus on the potential effect of azithromycin on the immune response to RILI, especially mechanisms that potentially could provide therapeutic benefit.

## 2 Pathophysiology of the radiation-induced lung toxicity

RILI in the early stage manifests as radiation-induced pneumonitis which occurs 1~6 months after radiotherapy while lung fibrosis (After radiotherapy 6~24 months) develops later (10).RILI occurs in nearly 30% of patients receiving high-dose radiation for therapy of lung cancer and a proportion of patients have symptomatic lung injury (11). The pathological mechanisms of RILI are complex and involve numerous cell types and signaling pathways (12). Previous studies have focused on radiation-induced vascular endothelial cell damage to further injury the alveolar-capillary barrier and reduce surfactant secretion from damaged alveolar-type cells (13, 14). Recently, studies have found lung macrophages as non-proliferative and highly differentiated natural immune cells, not only is there a certain tolerance to the radiation (15), but also it plays an important regulatory role in the whole pathological process of RILI (16). Lung macrophages promote the reactive oxygen-induced reactive oxygen cascade reaction, and the progress of the effects of inflammatory storm and the acceleration of fibrosis in RILI.

Radiation injury is a process which physics technology leads to biological change. In lung tissue it manifests the damage of various types of cells including lung epithelial. vascular endothelial cells, I and II type alveolar cells, organized residence macrophages, *via* the production of reactive oxygen species and reactive nitrogen species (ROS/RNS) and inflammatory cytokines by single-strand DNA breaks and indirect ionization of water moleculars. Normal alveolar structure is destroyed and alveolar barrier function is lost. Afterwards, alveolar and interstitial edema is formed, then inflammatory cells outside like macrophages and neutrophils are recruited and accumulated here to effect action. Above happen at early phase. after few months fibroblasts differentiate and participate in it which leads to chronic radiation injury (17, 18). Different sources of macrophages involving alveolar macrophages, interstitial macrophages and foreign macrophages all play important roles by polarizing different functional macrophages *via* various cytokines like interferon-beta (IFN-β) and Interleukin-4(IL-4) (19). At the initial stage of radiation injury, T helper cell type 1 (Th-1) cells were activated to release interferon-beta (IFN-β) stimulated M1 macrophage activation, meanwhile, Th-2 inflammatory cells were inhibited. When the injury continues to develop, Th-2-derived cytokines Interleukin-4 (IL-4) and IL-3 are released at the injury site to transform the injury into abnormal wound healing, it is characterized by the accumulation of M2 macrophages, which ultimately decrease the inflammatory process. Most medical treatments for RILI mainly choose to act during early phase in clinical practice (20, 21).

## 3 Immunomodulatory mechanisms of azithromycin

### 3.1 Azithromycin inhibits inflammatory cell signaling pathways

Azithromycin exerts an anti-inflammatory effect by inhibiting signaling pathways relevant with inflammatory responses. Previous studies demonstrated that azithromycin prevents the activation of nuclear translocation of NF-κB signaling pathway thereby reducing the up-regulation of pro-inflammatory gene expression (2, 22). These results also involve in the evaluation of the impact of azithromycin upon other aspects of inflammatory cell signaling including suppression of the inflammasome, and inhibition of phospholipase-A2 (PLA2) (23–25). In THP-1 human monocytic cells, azithromycin inhibits lipopolysaccharide (LPS)-induced macrophage-derived chemokine (MDC) expression through c-Jun N-terminal kinase (JNK) and NF-κB/p65 pathways. Azithromycin also inhibits the expression of LPS-induced IFN-inducible protein 10 (IP-10/CXCL10), which is a T helper (Th)1-related chemokine that causes asthma airway inflammation and hypersensitivity through the MAPK-JNK/ERK and NF-κB/p65 pathways (25). Decreases in NF-κB DNA binding site were mechanistically linked to the suppressed induction of pro-inflammatory genes and cytokine production in different murine and other models of inflammatory and infectious diseases *in vitro* (26). Many potential immunomodulatory effects of azithromycin have been reported including down-regulating prolonged inflammation, decreasing airway mucus secretion, inhibiting bacterial biofilm (27).

### 3.2 Azithromycin alters macrophage polarization

**Figure 1:**
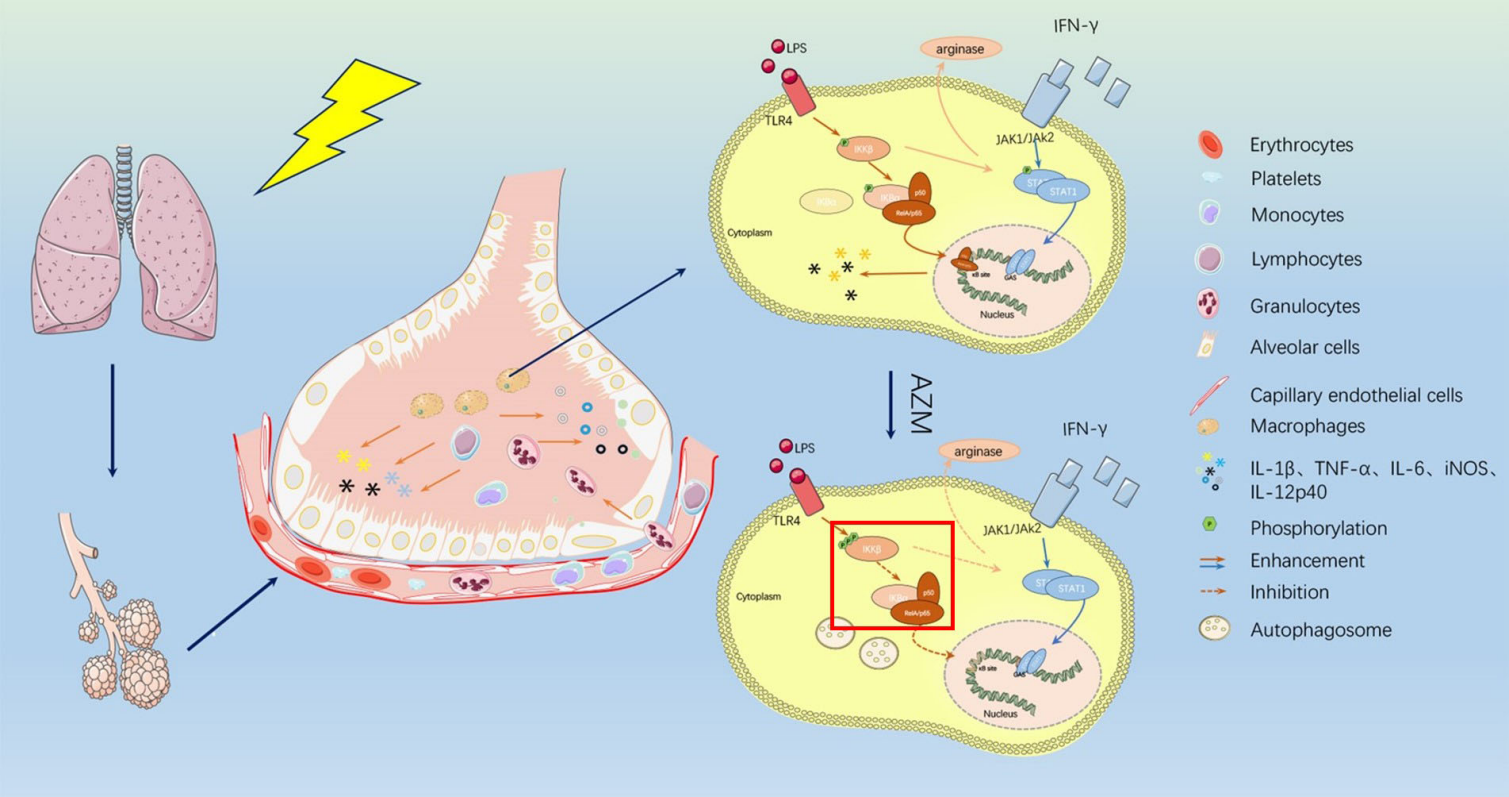
The mechanism of azithromycin in RILI patients. After ionizing radiation applied to lung tissue, alveolar barrier and vascular endothelial cells are damaged and vascular permeability is changed. Kinds of inflammatory cells like monocytes, lymphocytes, and macrophages are recruited here and released inflammatory cytokines such as IL-1beita, IL-6, iNOS, IL-12p40, TNF-a. Azithromycin can act on macrophages which made IKKb/NF-kB pathways suppressed. Azithromycin treatment increased the phosphorylation of IKKb resulting in inhibition of NF- kB translocation into the nucleus and resulting in inhibit signal transducer and activator of transcription-1(STAT-1) signaling. Both made inflammatory process affected and release of cytokines is decreased.

Macrophages play pro-inflammatory and anti-inflammatory roles through classical and alternative activation pathways, Which we refer to the polarized macrophages as M1 and M2 (28). The former is characterized by inducible nitric oxide synthase, and the latter is marked by arginase-1. M1 macrophages have been shown to participate in pro-inflammatory responses, and M2 macrophages are the main type of macrophages in pulmonary fibrosis. Based on the stage of the disease and its interaction with other immune cells, macrophages are polarized and exert anti or pro inflammatory reactions. Experiments using the murine macrophage cell line J774 considered that azithromycin can polarize macrophages to a M2 alternative-like phenotype *in vitro (* 29). In macrophages polarized to a M1 phenotype with IFN-γ and LPS, azithromycin inhibited pro-inflammatory cytokine expression (including IL-12 and IL-6) and shifted surface receptor expression from M1 phenotype to what typically observed in alternatively-activated macrophages. A recent study shed additional light on the mechanism by which azithromycin polarized macrophages to an alternative, anti-inflammatory phenotype (3). Incubation result of a murine macrophage cell line or primary murine macrophages with azithromycin was observed to increase the overall expression of IκB kinase (IKKβ), a molecule involved in signaling to NF-κB activation. When cells were stimulated with IFN-γ and LPS, azithromycin treatment increased the phosphorylation of IKKβ despite a reduction in the subsequent signaling resulting in inhibition of NF- κB translocation into the nucleus (3). A previous report explained the phenomenon that the over-expression of IKKβ can inhibit signal transducer and activator of transcription-1(STAT-1) signaling (the pathway responsible for classical macrophage activation in the presence of IFN-γ) (30), investigators then explored this connection and found that azithromycin inhibited the phosphorylation of STAT-1, which was dependent upon IKKβ. Induction of the M2 protein arginase was also dependent on this cross-talk, as IKKβ inhibitors reversed the ability of azithromycin to induce arginase activity (3). This work provides a possible mechanistic link between NF-κB signaling inhibition and macrophage polarization by the drug. **Figure 1** has shown the mechanism of azithromycin in RILI patients. According to different sources, we can divide macrophages into alveolar macrophages(AMs), interstitial macrophages(IMs) and infiltrating monocyte-derived macrophages. Kinds of macrophages play different functions in radiation-induced lung injury Lydia, et al’s study raised that IMs expressed 10-fold more arginase (Arg)-1 than alveolar macrophages (AMs), and a 40-fold upregulation of Arg-1 was found in IMs isolated from radiation lung fibrosis. IMs, but not AMs, were able to induce myofibroblast activation *in vitro* by clinical and preclinical research. It suggests us future study could focus on kinds of macrophages in different phases of RILI (31).Furthermore, the study from Hodge affirmed that Azithromycin has an anti-inflammatory properties by improved the phagocytosis of epithelial cells or neutrophils by AMs from COPD and decreases levels of pro-inflammatory cytokines are high doses (32).

After ionizing radiation applied to lung tissue, alveolar barrier and vascular endothelial cells are damaged and vascular permeability is changed. Kinds of inflammatory cells like monocytes, lymphocytes, and macrophages are recruited here and released inflammatory cytokines such as IL-1beita, IL-6, iNOS, IL- 12p40, TNF-a. Azithromycin can act on macrophages which made IKKb/NF-kB pathways suppressed. Azithromycin treatment increased the phosphorylation of IKKb resulting in inhibition of NF- kB translocation into the nucleus and resulting in inhibit signal transducer and activator of transcription-1(STAT-1) signaling. Both made inflammatory process affected and release of cytokines is decreased.

### 3.3 Azithromycin inhibits autophagosome clearance

Autophagy, as a cellular process induced in both physiological as well as pathophysiological conditions, is essential for cell survival to maintain a good balance between protein synthesis and degradation (33), and also plays a complex role in pathogen elimination and inflammatory regulation (34). At therapeutic concentrations, azithromycin was indicated to increase the number of macrophages autophagosomes (4, 5). This increase in number may be due to inhibiting the degradation of autophagosomes rather than increasing their synthesis (4). Azithromycin accomplishes this by inhibiting lysosomal acidification which thereby inhibits autophagosome clearance (4). Autophagy induction is considered to have an anti-fibrotic effect and can be modulated by drugs. The autophagy inducer rapamycin protects against bleomycin induced lung fibrosis, and impaired autophagosomes in RIPF may lead to fibrogenesis and promote fibroblast activation and extracellular matrix production (35). Recently, a study of interstitial pulmonary fibrosis has shown that autophagy is reduced after azithromycin treatment and it affects fibrosis (36). Furthermore, facilitation of autophagy flux has also been linked to increases in pathogen elimination (37). The association between autophagy and inflammation contributes to increase more comprehensive understanding of RILI (38), the impact of azithromycin at this nexus remains to be studied.

### 3.4 Azithromycin impacts neutrophils

Azithromycin can directly affect the function of neutrophils. Neutrophils stimulated by injury related molecular patterns and other signals, play important, though often destructive, roles in airway diseases including asthma, Chronic obstructive pulmonary disease (COPD), and ARDS. Azithromycin exhibits rapid and prolonged cellular accumulation and has a very long half-life in these cells (39). It is no doubts that azithromycin as an anti-fibrotic, inhibits inflammatory process. From Weronika et al’s study, the result indicated azithromycin regulated the pro-inflammatory ability of the neutrophils by decreasing respiratory burst and the release of neutrophil extracellular traps (NETs), which are as methods of neutrophils to kill pathogens (40). And another report also demonstrated that the macrolide erythromycin decreases airway NET formation in mice (41). Azithromycin has also been shown in pre-clinical studies to decrease IL-8 release and neutrophil airway infiltration, cause degranulation and degradation of extracellular myeloperoxidase, and reduce neutrophil oxidative burst (42). Indeed, azithromycin, frequently used in asthmatic children with lower respiratory tract infection, inhibits the accumulation of neutrophils in pulmonary airways by affecting interleukin-17 downstream signal, and by inhibiting the release of neutrophil mobilizing cytokines: macrophage inflammatory protein-2(MIP-2), CXC chemokine ligand-5(CXCL-5), and granulocyte macrophage colony-stimulating factor(GM-CSF) (43). In addition, azithromycin attenuates neutrophil function. It down-regulates chemical attractants and adhesion molecules in activated vascular endothelial cells, reduces neutrophil activation, and limits the release of NET (40).

## 4 The potential impact of azithromycin on RILI

**Figure 2:**
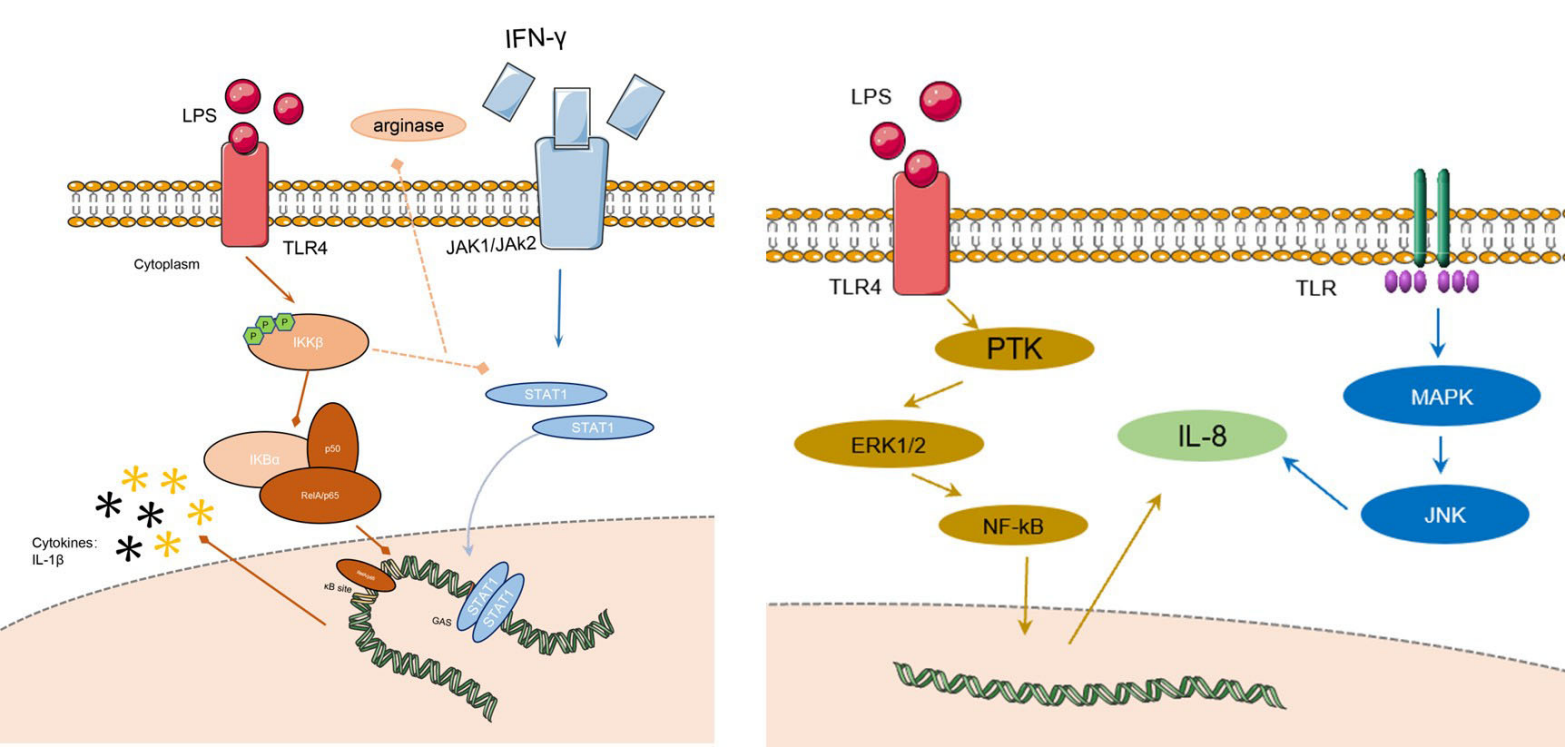
The signaling pathways of AZM on RILI. The left figure shows the changes of macrophage in action of AZM: the IkB subunit will not be degraded by the phosphorylation of IkKB, and will continue to bind to the NF-kB subunit, preventing the development of downstream pathways, finally the process inhibits the release of M1 phenotype inflammatory factors, and promotes M1-like phenotype macrophages to M2-like phenotype. The right figure shows that IL-8 could be regulated by many signaling pathways like ERK1/2/NF-kB, MAPK, JNK etc. LPS, lipopolysaccharide; IFN-g, interferon-g; TLR, toll-like receptor; MAPK, mitogen-activated protein kinase; JNK, c-Jun N-terminal kinase; IL-8, interleukin; NF-kB, nuclear factor kappa-B; ERK, extracellular regulated protein kinases; PTK, protein tyrosine kinase; JAK, just another kinase.

RILI is a common complication of radiation therapy. Development of an effective and sensitive drug that selectively decrease damage of normal lung tissues receiving radiation is an urgent question to be solved. Researchers focus on azithromycin on account of poly-pharmacological properties. The potential beneficial effects of azithromycin in RILI can be explained by some mechanisms (**Figure 2**), which includes killing pathogens, inhibiting the production of pro-inflammatory cytokines and inducing the regulatory function of macrophages (44). Thus, azithromycin may be a promising drug for the treatment of RILI which is attributed to its immunomodulatory properties and very high and stable lung concentrations (45). Firstly, azithromycin is an effective regulator of cytokines derived from monocytes and macrophages. It may inhibit the NF-κB signaling pathway to decrease inflammatory responses, and reduce the production of differentiation markers IL-6, IL-8 and tumor necrosis factor-alpha (TNF-α) from the classical M1 activated macrophages and the release of GM-CSF to balance the immune response after radiation. Secondly, azithromycin affects functions of macrophages. During the thoracic irradiation, macrophage polarization triggers inflammatory and immune cells activation and infiltration, leading to the occurrence of RIP and RIPF. The ultimate pathological consequences of RILI in lung tissues depends on the relative equilibrium and activity of M1/M2 macrophages (46). Azithromycin significantly attenuates the accumulation of M1 macrophages and modulates the polarization balance of macrophages to reduce the inflammatory process. Modifications in lung macrophages after radiation have been both detected during early and late stages of tissue injury, supporting the notion that azithromycin has important meanings in treatment of RILI by regulating macrophages. In addition, azithromycin also enhances host defense and controls inflammatory related damage by inhibiting neutrophil killing mechanisms and increasing the number of autophagosomes in macrophages. Furthermore, the infiltration or exudation of inflammatory cells into the lung parenchyma seems to play an important role in the development of RILI. Preliminary evidence suggests that pharmacological or other interventions may be possible to reverse the manifestation of the injury and restore function to lung tissues (47).

Currently, the non-antibacterial inflammatory and immunomodulatory effects of AZM have been demonstrated in a variety of diseases, such as COPD, asthma, cystic fibrosis, and idiopathic pulmonary fibrosis (48). Affirming the inflammatory and immunomodulatory effects of AZM, its use in the treatment of radiation pneumonitis became possible. Based on the above-mentioned occurrence of radiation pneumonitis and the mechanism of action of AZM in many aspects, there may be unexpected effects on the treatment of radiation pneumonitis by utilizing the effect of AZM.

### 4.1 IL-8/MAPK/ERK/neutrophils signaling pathway

IL-8 acts as a neutrophil chemokine that promotes neutrophil migration to sites of inflammation. IL-8 levels are elevated in patients with radiation pneumonitis, thus, which could be a potential predictive marker for radiation pneumonitis. Early intervention of IL-8 may be effective in reducing the occurrence of radiation pneumonitis. Previous studies have reported that IL-8 has a dose-dependent relationship with AZM. Short-term use of AZM can lead to an increase in IL-8 while IL-8 levels will decrease after 5 days of continuous use. The mitogen-activated protein kinase (MAPK) pathway, extracellular regulated protein kinase (ERK), c-jun NH2-terminal kinase (JNK), and the p38 MAPK cascade contribute to IL-8 gene expression (49). Control of IL-8 can be achieved through the use of MAP kinase/ERK kinase inhibitors, thereby reducing neutrophil chemotaxis (50).

### 4.2 IL-1β/STAT-1/NF-kB/M2 macrophages signaling pathway

In the early stage of radiation-induced lung injury, macrophages in lung tissue showed M1-like phenotype and released pro-inflammatory factors, such as IL-1β, IL-6, TNF-α, etc., to create an inflammatory microenvironment. AZM can mediate the STAT-1/NF-kB pathway in the early stage of RP to polarize macrophages in the M1-like phenotype to the M2-like phenotype, thereby reducing the degree of inflammation. The details show in **Figure 1**. Under the action of TLR and IL-1, IκB subunit bound to the dimer subunit P50/P65 of NF-kB was ubiquitinated and degraded in M1 macrophages. The combination of IκB subunit with P50/p65 can prevent p50/p65 was phosphorylated to activate downstream signaling pathway and prevent its further transcription into the nucleus to bind to the NF-kB DNA promoter region, which could control the expression of proinflammatory cytokines and related genes. After the action of AZM, the IκB subunit will not be degraded by the phosphorylation of IκKB, and will continue to bind to the NF-κB subunit, preventing the development of downstream pathways, finally the process inhibits the release of M1 phenotype inflammatory factors, and promotes M1-like phenotype macrophages to M2-like phenotype. Based on this, the early inflammation of radiation pneumonitis can be alleviated (3). The action of AZM on NF-kB has been reported in many studies to increase the credibility of the pathways (51, 52).

### 4.3 The application of AZM on RILI

AZM’s research on RILI has been reported in the literature. In a mouse model of radiation pneumonitis, the application of AZM can significantly reduce the levels of various inflammatory or fibrotic factors such as IL-1β, IL-6, TNF-α, TGF-β1 in bronchoalveolar lavage fluid (BAL) and plasma. It also decreased the expression levels of fibrosis marker mRNAs. AZM has a certain inhibitory effect on oxidative stress and inflammatory response in the pathogenesis of radiation pneumonitis. The oxidative damage marker malondialdehyde (MDA) is also significantly decreased after the utilization of AZM, and the number of inflammatory cells in BAL is reduced, and the inflammatory cells in BAL decrease. Furthermore, researchers found the effect was more significant in the high-dose group (100 mg/kg/day) (9). The clinical treatment results of macrolides for radiation pneumonitis show that the application of macrolides like clarithromycin can reduce the incidence of any grade and high-grade radiation pneumonitis in patients, and has a high risk of radiation pneumonitis. Patients with high risks of radiation pneumonitis factors (such as idiopathic interstitial pneumonia (IIP)) had better treatment effects. The clinical outcome was observed that IL-8 and total cell counts in BAL of RP patients treated with clarithromycin were significantly decreased (7). There is no clinical research report of AZM on radiation pneumonitis, but based on the therapeutic mechanism of macrolides and the pathogenesis of RILI, AZM has a great research value for the treatment of RILI. Despite its promising outcome, the complications of AZM should be noticed. For example, the use of AZM should be closely monitored in patients with pre-existing cardiac problems, arrhythmias, baseline QT prolongation, and electrolyte disturbances (8).

## Conclusion

Pleiotropic mechanisms of azithromycin have raised a large interest in treating RILI from immune and inflammatory aspects. However, the immunomodulatory effects of azithromycin are complex and multifactorial, including affecting macrophage polarization, neutrophils influx, and cytokines release, and lack of direct proof for RILI treatment. Whatever, any treatment that impacts immune function should be carefully applied for patients with a risk of infection. Immune regulatory function of azithromycin needs to be further explored in the setting of RILI, and it is worth noting that basic information of patients should be reasonably evaluated before utilizing azithromycin in clinical practice.

Cardiac problems, arrhythmias, baseline QT prolongation, and electrolyte disturbances (8).

## Author contributions

YY and LW were major contributors in writing the manuscript. All authors contributed to the article and approved the submitted version.
